# Morphological and functional alterations in adult boar epididymis: Effects of prenatal and postnatal administration of flutamide

**DOI:** 10.1186/1751-0147-53-12

**Published:** 2011-02-22

**Authors:** Marta Lydka, Ilona Kopera-Sobota, Malgorzata Kotula-Balak, Katarzyna Chojnacka, Dorota Zak, Barbara Bilinska

**Affiliations:** 1 Department of Endocrinology, Institute of Zoology, Jagiellonian University, Krakow, Poland

## Abstract

**Background:**

The dynamic cross-talk between epididymal cells is hormonally regulated and, in part, through direct cell-to-cell interactions. To date, no information is available regarding possible impact of anti-androgens on the proteins involved in the gap junctional communication within the boar epididymis. Thus, a question arised whether prenatal or postnatal exposure to an anti-androgen flutamide alters the expression of gap junction protein - connexin43 (Cx43) and androgen receptor (AR) expression in the caput, corpus and cauda epididymis and leads to delayed effects on morphology and function of adult pig epididymis.

**Methods:**

First two experimental groups received flutamide prenatally on gestational days 20-28 and 80-88 (GD20 and GD80) and further two groups were exposed to flutamide postanatally on days 2-10 and 90-98 after birth (PD2 and PD90). Epididymides were collected from adult boars. Routine histology was performed using hematoxylin-eosin staining. The expression of Cx43 and AR were analyzed using immunohistochemistry and Western blotting. Both analyses were supported by quantitative approaches to demonstrate the variations of the expression levels following the treatment. Apoptotic cells were identified using TUNEL assay.

**Results:**

Histological examination revealed differences in epididymal morphology of flutamide-exposed boars when compared to controls. Scarce spermatic content were seen within the corpus and cauda lumina of GD20, PD2 and PD90 groups. Concomitantly, frequency of epididymal cell apoptosis was significantly higher (*p *< 0.05) after exposure to flutamide at GD20. Moreover, in GD20, PD2, and PD90 groups, significantly lower AR expression (*p *< 0.05) was found in the principal and basal cells of the corpus and cauda regions, while in the stromal cells AR expression was significantly reduced (*p *< 0.05) along the epididymal duct. Concomitantly, a decrease in Cx43 expression (*p *< 0.05) was noticed in the stromal cells of the cauda region of GD20 and PD2 groups. This indicates high sensitivity of the stromal cells to androgen withdrawal.

**Conclusions:**

The region-specific alterations in the epididymis morphology and scarce spermatic content within the lumina of the corpus and cauda indicate that flutamide can induce delayed effects on the epididymal function of the adult boar by decrease in AR protein levels that results in altered androgen signaling. This may cause disturbances in androgen-dependent processes including Cx43 (de)regulation, however, we can not exclude the possibility that in response to flutamide decreased Cx43 expression may represent one mechanism responsible for functional disturbance of the boar epididymis.

## Background

The intercellular communication mediated by gap junctions coordinates cellular functions within reproductive tissues and whole organs in a complementary way to other regulation of basic cellular functions [1]. Throughout the length of the ductus epididymis communication systems are based on a secretion of hormones [2]. Their cooperation determines the appropriate luminal microenvironment that is formed for the functional maturation, storage and protection of spermatozoa [3,4]. Due to several mechanisms various proteins secreted in the epididymis are highly regionalized and are specific to each region of the adult boar [5]. Although gap junctions between adjacent principal cells of epididymis were identified by freeze fracture electron microscopy almost forty years ago [6] the presence and the physiological role of gap junctions in the processes that involve the final maturation of spermatozoa during their transit along the epididymal duct are largely unknown. Scarce information is also available regarding possible effects of anti-androgens on the integral proteins that build intercellular junctions in the male [7,8].

Since androgens are known to maintain the morphology and secretory function of the epididymis acting directly through androgen receptor (AR) [9-11], the effect of an antiandrogen flutamide, which is known to hinder androgen action by inhibiting receptor binding of androgen is of special interest. It should be mentioned that Pearl et al. [11] demonstrated no change in the concentration of androgens and estrogens throughout the boar epididymis and suggested that regional differences in steroid regulation are likely due to differences in receptor expression. The effects of androgen deprivation induced by orchiectomy, GnRH agonist administration and ethane dimethane sulphonate treatment have been shown in the epididymis of several mammalian species including pigs [12-15]. Recently, changes in the expression of antioxidant enzymes that protect germ cells from damage by various forms of reactive oxygen species have been demonstrated in epididymal epithelial cells of finasteride-treated rats [16]. Androgen receptors have been localized to epididymal cells in humans and several other mammalian species [17-20], however, the effect of flutamide on AR expression has never been investigated in porcine epididymis.

The question arises whether adverse effects of flutamide on epididymal morphology and function can be related either to affected gap junction communication and/or altered expression of the AR.

To show a role of androgens in the modulation of the gap junctional intercellular communication and sensitivity of epididymal cells to androgens, the expression of predominant gap junction protein connexin 43 (Cx43) and the AR were detected in three regions of adult boar epididymis by means of immunohistochemistry and Western blotting. To check whether the effect of flutamide depends on the time of its administration, prenatal and postnatal exposures were applied. It should be added that recently we have demonstrated that exposure to flutamide induced adverse effects on male and female gonad morphology and the expression of Cx43 protein in gonads of prepubertal pigs [21], not in neonates [22].

## Materials and methods

### Animals and experimental design

Nine-month-old adult boars (Large White × Polish Landrace) originating from five litters were allotted into four groups of experimental animals (n = 3) and respective control groups (n = 3). First two experimental groups were exposed prenatally on gestational days 20-28, and 80-88 (GD20 and GD80) to an anti-androgen flutamide (2-methyl-N-[4-nitro-3-(trifluoromethyl)-phenyl]propamide; Sigma-Aldrich, St Louis, MO, USA). Two further groups were treated with flutamide postanatally on days 2-10 and 90-98 after birth (PD2 and PD90). The control animals were given a vehicle only (corn oil). Flutamide was administered at a dose of 50 mg/kg body weight five times every second day. The flutamide exposure was based on the literature [23] and our own data described in detail previously [21,22].

The epididymides were obtained from 270-day-old animals, irrespective of the time of flutamide treatment. All surgical procedures were performed by a veterinarian and followed approved guidelines for the ethical treatment of animals in accordance with the Polish legal requirements under the licence given by the Local Ethics Committee at the Jagiellonian University, Krakow, Poland (No. 4/2008).

### Tissue Preparation

Every two epididymides of both control and flutamide-treated boars (n = 3 each group) were surgically removed and small fragments of the epididymal tissue were immersed either in Bouin fixative or in 4% formaldehyde freshly prepared from paraformaldehyde for histology or immunohistochemistry, respectively, and embedded in paraplast (Monoject Scientific Division of Scherwood Medical, St Louis, MO). Sections of 6 μm in thickness were mounted on slides coated with 3-aminopropyltriethoxysilane (APES; Sigma-Aldrich Chemical Co., St Louis, MO), deparaffinized and rehydrated. For routine histology haematoxylin-eosin (H-E) staining was performed.

### Immunohistochemistry

To optimize immunohistochemical staining slices were immersed for 2 × 5 min in 10 mM citrate buffer (pH 6.0) and heated in the microwave oven (750 W). Details of a whole procedure have been described elsewhere [24,25]. Briefly, nonspecific staining was blocked twice, first with 3% H_2_O_2 _in methanol for 15 min, to inhibit endogenous peroxidase activity, and second with 5% normal goat serum for 30 min at room temperature to block nonspecific binding sites. Thereafter, sections were incubated overnight at 4°C in a humidified chamber in the presence of polyclonal antibodies against Cx43 (1:2000; Sigma-Aldrich; St Louis, MO, USA) and AR (1:2000; Santa Cruz Biotechnology, Santa Cruz, CA, USA). Next, biotinylated secondary antibody, goat anti-rabbit IgG (1:400; Vector, Burlingame CA, USA) was applied. Finally, avidin-biotinylated horseradish peroxidase complex (ABC/HRP; 1:100; Dako, Glostrup, Denmark) was used. After each step in these procedures, sections were carefully rinsed with Tris-buffered saline (TBS; 0.05 M Tris-HCl plus 0.15 M NaCl, pH 7.6); the antibody was also diluted in TBS buffer. Bound antibody was visualized with TBS containing 0.05% 3.3'-diaminobenzidine tetrachloride (DAB), 0.01% H_2_O_2 _and 0.07% imidazole for 3-4 min. Counterstaining with Mayer's hematoxylin was additionally performed. In control sections, the primary antibody was replaced by normal goat serum. Then, sections were examined with a Leica DMR microscope (Leica Microsystems, Wetzlar, Germany) using a bright field illumination for morphology and Nomarski interference contrast for immunohistochemistry.

### Qualitative and quantitative evaluation of the immunohistochemical reactions

Immunohistochemical staining for both antigens, Cx43 and AR, was evaluated qualitatively in at least 20 serial sections of epididymides from each experimental group. The slides were processed immunohistochemically at the same time with the same treatment so that the staining intensities could be compared. The epididymal cells were considered immunopositive if brown reaction product was present in the cell nuclei (AR) or appeared as signal between epididymal cells (Cx43); the cells without any specific immunostaining were considered immunonegative.

To evaluate the intensity of immunohistochemical reaction quantitatively, digital color images were obtained using a CCD Video Camera (KY-F55, JVC) mounted on an optical microscope (Microphot, Nikon, Japan) and connected to a video capture card (PV-BT878P, Prolink, Taiwan) installed on a personal computer. Images of the epididymides were captured using a 20 × objective as described previously [26]. Image processing and analyses were performed using the public domain ImageJ software (National Institute of Health, Bethesda, MD, USA). The intensity of the immunohistochemical reaction was expressed as relative optical density (ROD) of diaminobenzidine brown reaction product and calculated using the formula described by Smolen [27]. A total number of 50 epididymal sections (n = 10 per group) were subjected to image analysis and results of 10 separate measurements were expressed as mean ± SD.

### TUNEL assay

The presence of apoptosis-related DNA strand breaks in epididymal cells was evaluated by TUNEL assay using the In situ Cell Death Detection kit, POD (Roche Molecular Biochemicals, Mannheim, Germany) according to the manufacturer's instructions with our own modifications [28]. In short, after deparaffinization and rehydration, the 6 μm paraplast tissue sections were immersed in 3% H_2_O_2 _to quench endogenous peroxidase activity, rinsed in PBS and incubated with 10 μg/ml Proteinase K solution (Promega Corporation, Madison, WI, USA) for 15 min at 37°C. Thereafter, sections were incubated with TUNEL reaction mixture (terminal deoxynucleotidyl transferase and labeled nucleotide mixture). Next, sections were rinsed in PBS and incubated with Converter-POD (anti-fluorescein antibody conjugated with horse-radish peroxidase). Peroxidase activity was then visualized by precipitation of DAB (DAB Substrate, Roche, Mannheim, Germany). Cells containing fragmented nuclear chromatin exhibited a brown nuclear stain. As negative controls, sections were processed without terminal deoxynucleotidyltransferase (TdT) buffer. Apoptotic cells were examined with Leica DMR microscope. The number of TUNEL-positive cells was noted per 100 cross-sections of epididymal ducts.

### Western blot analysis

Tissues were homogenized on ice with a cold Tris/EDTA buffer (50 mmol/L Tris, 1 mmol/L EDTA, pH 7.5), sonicated and centrifuged at 10 000 × *g *for 20 min at 4°C as described previously [29]. In short, the protein concentration for each sample was estimated using Bradford dye-binding procedure with BSA as a standard [30]. Homogenates containing 50 μg of protein were solubilized in a sample buffer (Bio-Rad Labs, GmbH, München, Germany) and heated at 99.9°C for 3 min. After denaturation the samples were subjected to electrophoresis on SDS-PAGE gels (8% and 12%, v/v) according to Laemmli [31]. Separated proteins were transferred onto a nitrocellulose membrane using a wet blotter in the Genie Transfer buffer (pH 8.4) for 90 min at 250 mA. Then, blots were blocked overnight at 4°C with shaking in a solution of non-fat dry milk (5%, w/v) in TBST, followed by incubation with rabbit polyclonal antibodies against Cx43 (1:8000; Sigma-Aldrich) and AR (1:200; Santa Cruz) for 1.5 h at room temperature. The membranes were washed and incubated with a goat anti-rabbit IgG linked to the horseradish-peroxidase (1:1000; Vector Lab.) for 1 h at room temperature. Then, bound antibody was revealed using 3, 3'-diaminobenzidine as the substrate (DAB; 0.5 mg/mL). Finally, the membranes were dried and then scanned using Epson Perfection Photo Scanner (Epson Corporation, CA). Molecular masses for the AR and Cx43 were estimated by reference to standard proteins (Fermentas, GmbH, St. Leon-Rot, Germany and Bio-Rad Labs, Inc., CA USA), respectively. For positive controls pig prostate and pig heart were used. To obtain quantitative results, the bands representing each data point were densitometrically scanned using Image LaB™ 2.0 (Bio-Rad Labs). Quantitative analysis was performed for three separately repeated experiments from each control and experimental groups. Protein level within a control group was arbitrarily set as 1, against which statistical significance was analyzed.

### Statistical Analysis

All statistical analyses were performed using one-way analysis of variance (ANOVA) followed by Tukey's *post hoc *comparison test. The analysis was made using Statgraphics Centurion XV software. Data were presented as mean ± SD. The significance level was considered to be *p *< 0.05.

## Results

### Histological examination

**Figure 1:**
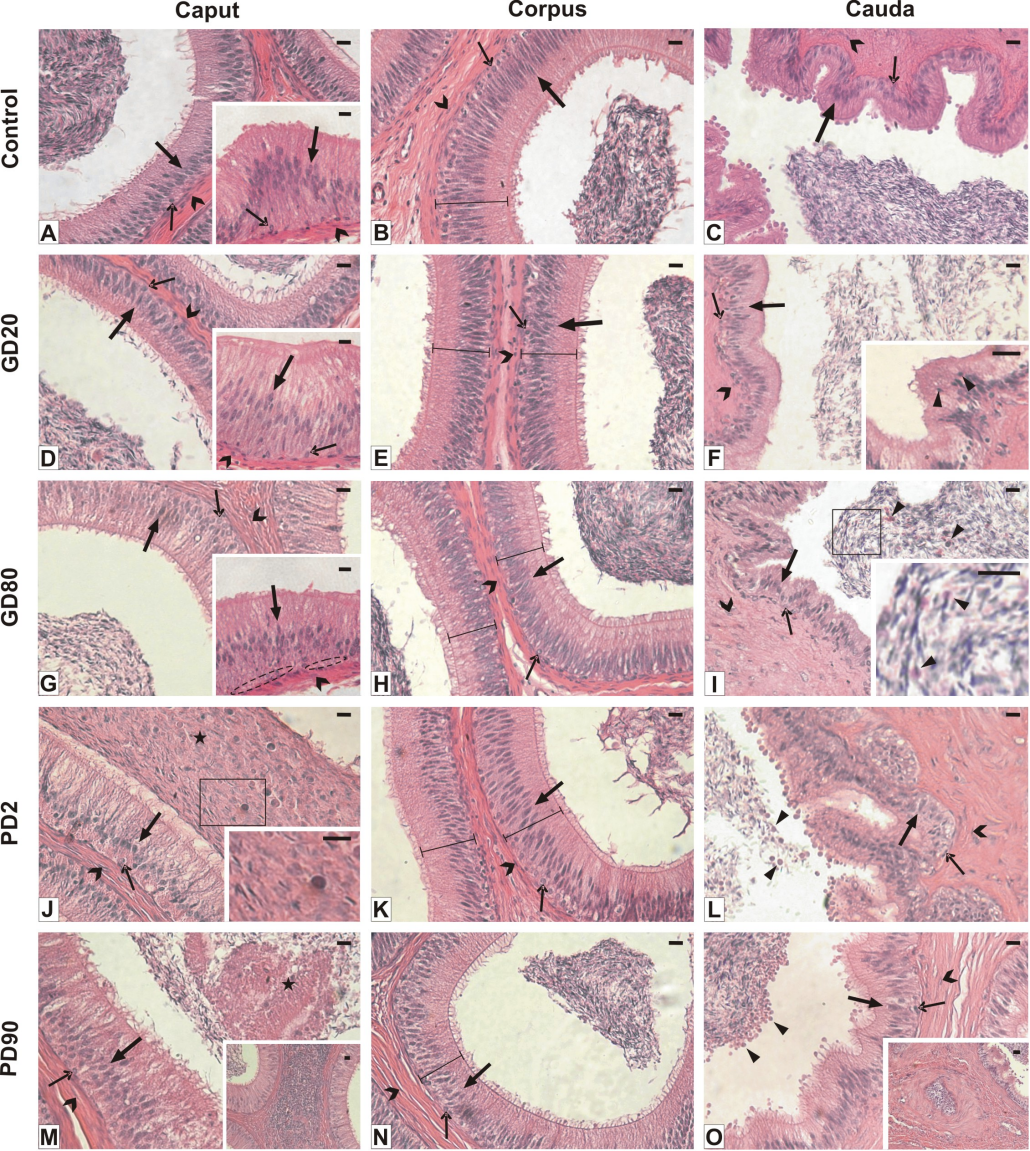
**Representative micrographs show morphology of epididymides of control (A-C) and flutamide-treated boars (D-O). Hematoxylin and eosin staining**. Scale bars represent 20 μm. Caput epididymis. In the control group **(A) **and flutamide-treated groups **(D, G, J, M) **the epithelium composed of principal cells (bold arrows) and basal cells (arrows) is observed. Outside the stroma layer is seen (open arrowheads). Irrespective of the groups, principal cells (bold arrow in inserts in **A, D, G**), few basal cells (arrow in inserts in **A, D**) or no basal cells (dashed-line ovals in insert in **G**) and stromal cells (open arrowhead in inserts in **A, D, G**) are seen in the initial segment of the epididymis. In PD2 and PD90 groups an agglomeration of phagocytic leukocytes in the lumen (asterisks) and sperm embedded in intraluminal cell debris are observed (**J, M**, at higher magnification, insert in **J**). Leukocytes in the stroma are also seen (insert in **M)**. Corpus epididymis. The composition of the cells in the epithelium and in the stroma layer is similar to that of the caput. Note a reduction of the epithelial heights (brackets) in males after flutamide treatment **(E, H, K, N) **compared to the control **(B)**. Scarce spermatic content are seen in PD2 and PD90 groups **(K, N) **compared to the respective controls **(J, M)**. Cauda epididymis. Note increased height of the epithelium and enlarged stroma layer after flutamide treatment **(F, I, L, O) **compared to the control **(C)**. In GD20 and GD80 groups less folded wall of the epididymal duct is seen **(F, I) **whereas in PD2 and PD90 males deep invagination of the epithelium **(L) **or even its closure is observed (insert in **O**). In lumina cell sloughing is seen (arrowheads) **(I**, insert in **I**) or bubble-like vesicles of sloughed cytoplasmic membranes are visible (arrowheads) **(L, O)**. After flutamide exposure at GD20 the intra-epithelial lymphocytes are seen (arrowheads) (insert in **F**). Scarce spermatic content are seen in GD20, PD2 and PD90 groups **(F, L, O) **compared to the respective controls (**D, J, M)**.

In both flutamide-treated and untreated boars the epididymal epithelium of the caput, corpus and cauda epididymis was composed of two major cell types: principal cells that outnumbered all the other cell types and basal cells. Independently of the group of boars, in the caput and corpus epididymis the principal cells were columnar in shape and became slightly lower in the cauda region (Figure 1A-O). The oval shaped basal cells were found throughout the epididymis besides the initial segment in which only few basal cells were present or they were not present at all (inserts in A, D, G). Spermatozoa were observed in the epididymal lumina of control and experimental groups (Figure 1A-O). In the latter, however, scarce spermatic content were seen in the corpus and cauda of GD20, PD2 and PD90 groups (Figure 1F, K-L, N-O). Concomitantly, the epithelial height of the cauda region increased and the stroma layer enlarged distinctly (Figure 1I, L, O). In the corpus, however, the epithelial heights were reduced (Figures. 1E, H, K, N). Frequently, the caudal epididymal region of GD20 and GD80 groups exhibited less folded appearance (Figure 1F, I), while that of the PD2 and PD90 showed deep invagination of the epididymal epithelium that sporadically led to its closure (Figure 1L, insert in O). In epididymal lumina either cell sloughing was observed (Figure 1F, I, insert in I) or bubble-like vesicles of sloughed cytoplasmic membranes were detected (Figure 1L, O). In the cauda of the GD20 group the intra-epithelial lymphocytes were frequently observed (insert in F). Occasionally, likely phagocytic leukocytes, characteristic of an inflammation response to tissue damage were seen either in the lumen or in the stroma of the caput epididymis of postnatally exposed boars (Figure 1J, M, inserts in J, M).

### TUNEL assay

**Figure 2:**
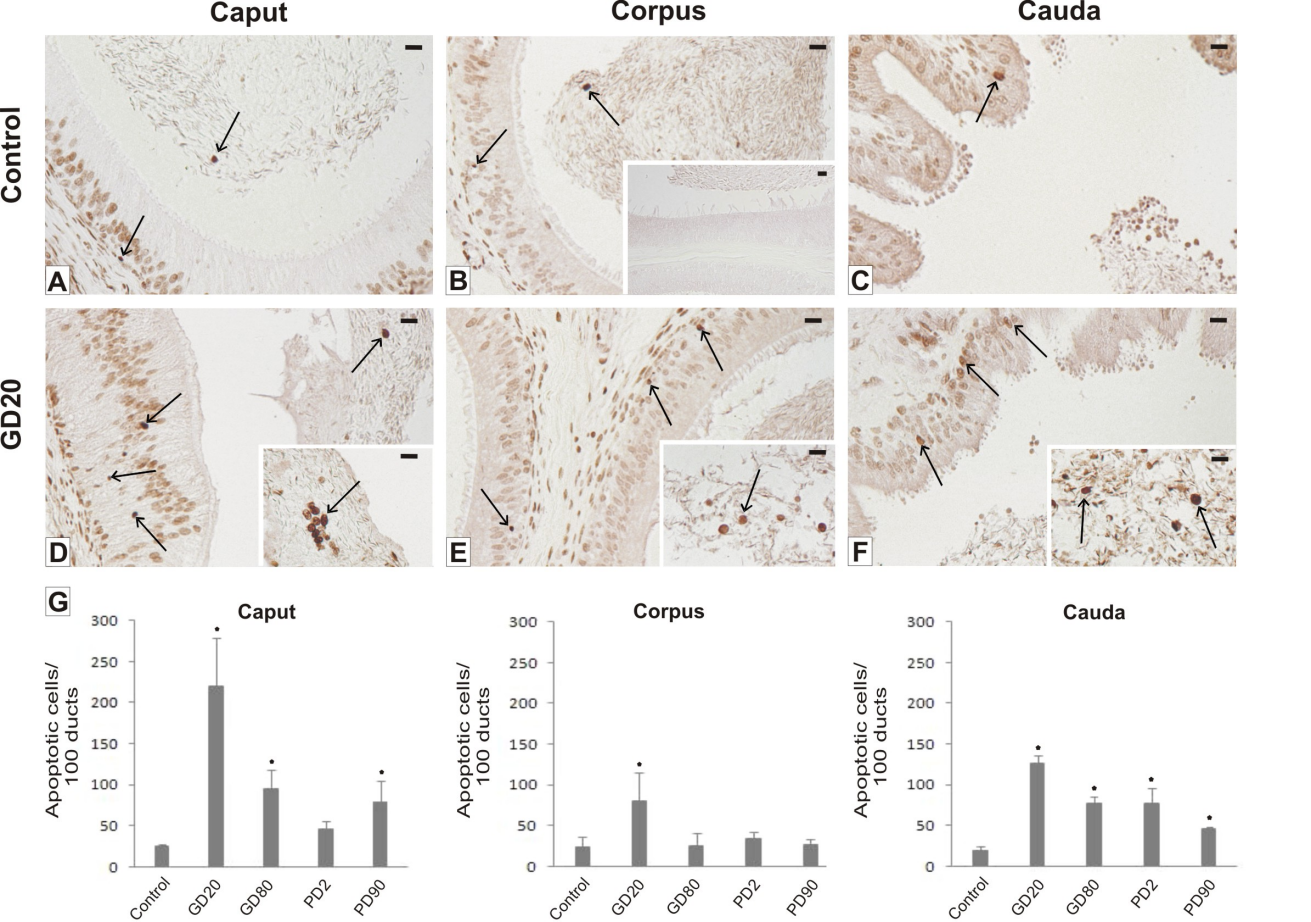
**Qualitative and quantitative analysis of apoptotic cells visualized using the in situ death detection kit POD**. Representative micrographs **(A-F) **and a histogram **(G) **of apoptotic cell number. Note increased number of apoptotic epithelial cells **(D-F) **and germ cells (inserts in **D-F**) along the epididymis of the GD20 group. Apoptotic cells are pointed out by arrows. Negative control sections, processed without TdT buffer, showed no staining (insert in **B**). Scale bars represent 20 μm. Values represent the mean ± SD number of TUNEL-positive germ cells per 100 epididymal duct sections. Significant differences from control values are denoted as *p < 0.05 (n = 3 for each group).

TUNEL staining was used to quantify apoptosis along the epididymal duct. As a result TUNEL-labeled cells were detected in epididymides of control (Figure 2A-C) and experimental groups, however the most prominent effect of flutamide, manifested by the apparent increase of apoptotic cell number, was noticed in the GD20 group (Figure 2D-F). Apoptotic cells were mostly accounted for as degenerating epithelial cells or germ cells transported from the testes after their degeneration. Microscopic evaluation was confirmed by quantitative analysis of the TUNEL assay that revealed significantly higher frequency of epididymal cell apoptosis (*p *< 0.01) especially in the caput and cauda regions of all groups examined when compared to the control (Figure 2K). In the GD20 group, mean numbers of TUNEL-positive cells per 100 cross-sections of epididymal ducts increased drastically in the caput (~9-fold), in the corpus (~3-fold), and in the cauda (~5-fold). Negative control sections, processed without TdT buffer, showed no staining (see, insert in Figure 2B).

### Qualitative and quantitative evaluation of Cx43 localization and expression

**Figure 3:**
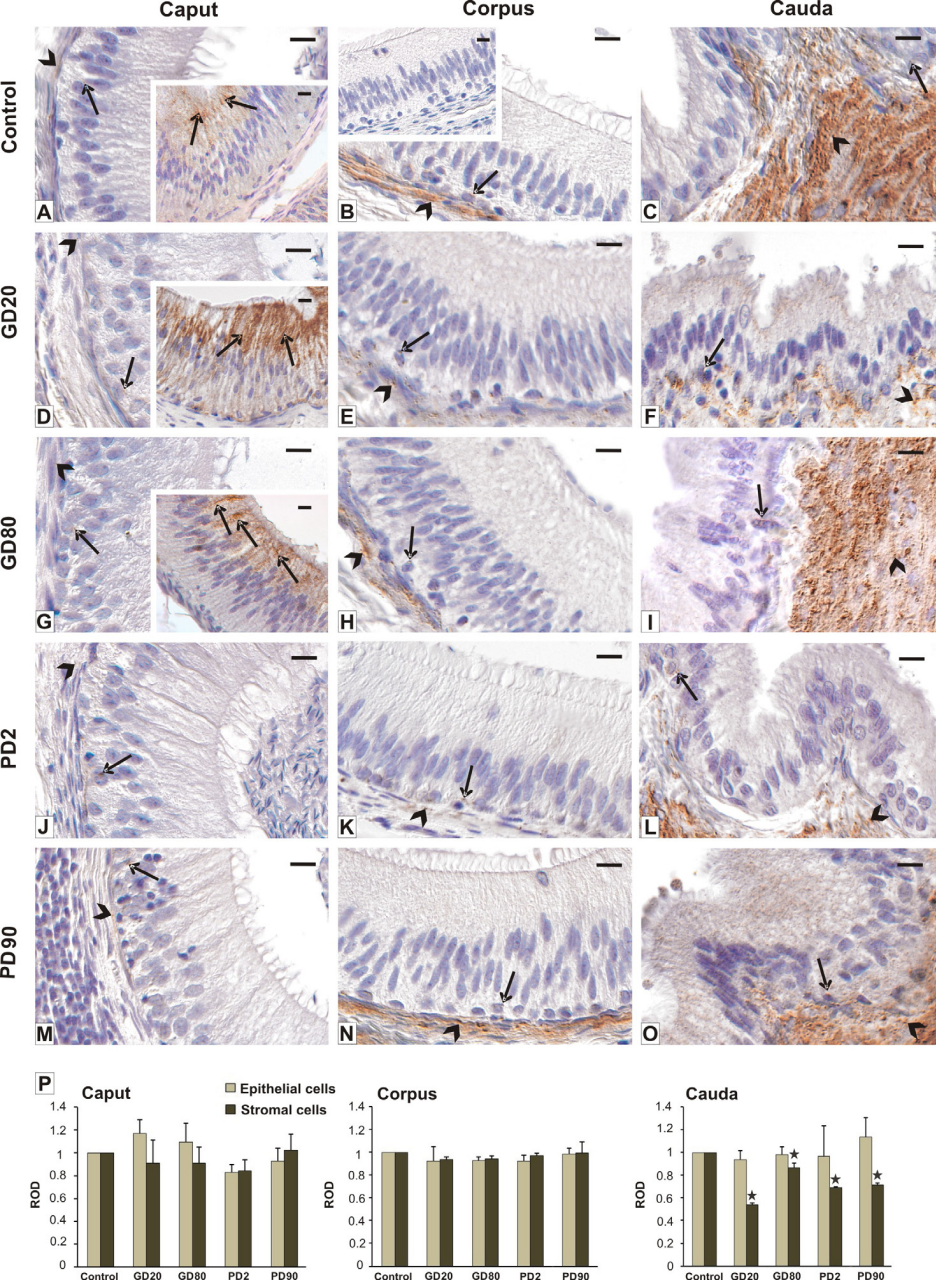
**Qualitative and quantitative analysis of immunohistochemical staining for Cx43**. Representative micrographs of control **(A-C) **and flutamide-treated boars **(D-O) **and a histogram of Cx43 staining intensity expressed as relative optical density (ROD) of diaminobenzidine brown reaction products **(P)**. Caput epididymis. In the control group **(A) **and flutamide-treated groups **(D, G, J, M) **very weak signal for Cx43 or no Cx43 signal is seen between the basal and principal cells (arrow) and between the basal and stromal cells (arrowhead). Note a strong signal for Cx43 between neighboring principal cells in the initial segment of the epididymis (arrows) of GD20 and GD80 groups *versus *control group (inserts in **A**, **D, G**) Corpus epididymis. In the control group **(B) **very weak, punctate signal between the basal and principal cells (arrow) and moderate to strong signal between adjacent stromal cells are seen. In GD20, GD80 and PD2 a slight decrease in Cx43 signal between stromal cells or no decrease (PD90) compared to control is seen, respectively **(E, H K, N) **Cauda epididymis. In the control group **(C) **strong signal for Cx43 between stromal cells (arrowhead) and weak signal among basal cells (arrow) are seen. Note a significant decrease in the signal intensity in stromal cells of GD20 and PD2 groups **(F, L)**, not in those of GD80 and PD90 **(I, O)**. Negative control sections processed without primary antibody showed no staining (insert in **B**). principal epithelial cells - large arrows, basal epithelial cells - arrows, stromal cells - arrowheads. Nomarski interference contrast. Bar represents 20 μm. Values are means ± SD. Asterisks indicate significant differences between the respective control group and groups treated with flutamide prenatally (GD20, GD80) and postnatally (PD2, PD90) (n = 3 for each group). Statistical significance: *p < 0.05

After flutamide exposure, the pattern of Cx43 staining was similar to the control, while the intensity of the staining varied in comparison with the control (Figure 3A-O). Irrespectively of the group of boars, very weak signal for Cx43 or no Cx43 signal was observed between the basal and principal cells and between the basal and stromal cells of the caput region (Figure 3A, D, G, J, M). Interestingly, in the initial segment an apparent signal for Cx43 was noticed between neighboring principal cells, especially of GD20 and GD80 groups in comparison to the control (inserts in A, D, G). In the corpus a very weak punctate signal was observed among the basal cells, while it was of moderate intensity between adjacent stromal cells (Figure 3B). Between the latter the signal decreased distinctly in GD20 and PD2 groups (Figure 3E, K). In the cauda of GD20 and PD2 groups a marked decrease in Cx43 immunoreactivity concerned the stromal cells (Figure 3F, L). No positive staining for Cx43 was detected when the primary antibody was omitted (insert in B). Qualitative analysis of Cx43 immunoreactivity (Figure 3A-O) was confirmed by a quantitative image analysis in which the signal intensities were expressed as relative optical density of diaminobenzidine deposits (Figure 3P). Statistically significant differences (*p *< 0.05) were found only in the stromal cells of the cauda region of GD20, PD2, and PD90 groups. For details, see Figure 3P.

**Figure 4:**
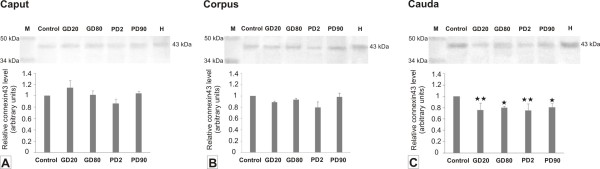
**Western blot analysis of Cx43 protein expression levels in epididymides of control and flutamide-exposed boars**. Representative Western blot shows a band at approximately 43 kDa in epididymal samples of control and flutamide-treated groups (prenatal exposure - GD20, GD80; postnatal exposure - PD2, PD90). Lane H indicates pig heart used as the positive control. Lane M contains prestained protein standards; the molecular weights in kDa are indicated on the left. Relative Cx43 levels in the caput, corpus and cauda epididymides **(A, B, C) **expressed as arbitrary units. Data obtained from three separate analyses is expressed as mean ± SD (n = 3 for each group). Significant differences from control values are denoted as *p < 0.05, **p < 0.01.

Using Western blot analysis bands at approximately 43 kDa were detected in epididymal samples of control and experimental groups (Figure 4A-C). Statistically significant changes in the Cx43 protein expression levels were demonstrated only in the cauda epididymis (*p *< 0.01) of GD20 and PD2 and (*p *< 0.05) of GD80 and PD90 groups what confirmed immunohistochemical findings, pointing out that flutamide treatment had a region-specific effect on Cx43 protein expression at adulthood.

### Qualitative and quantitative evaluation of AR localization and expression

**Figure 5:**
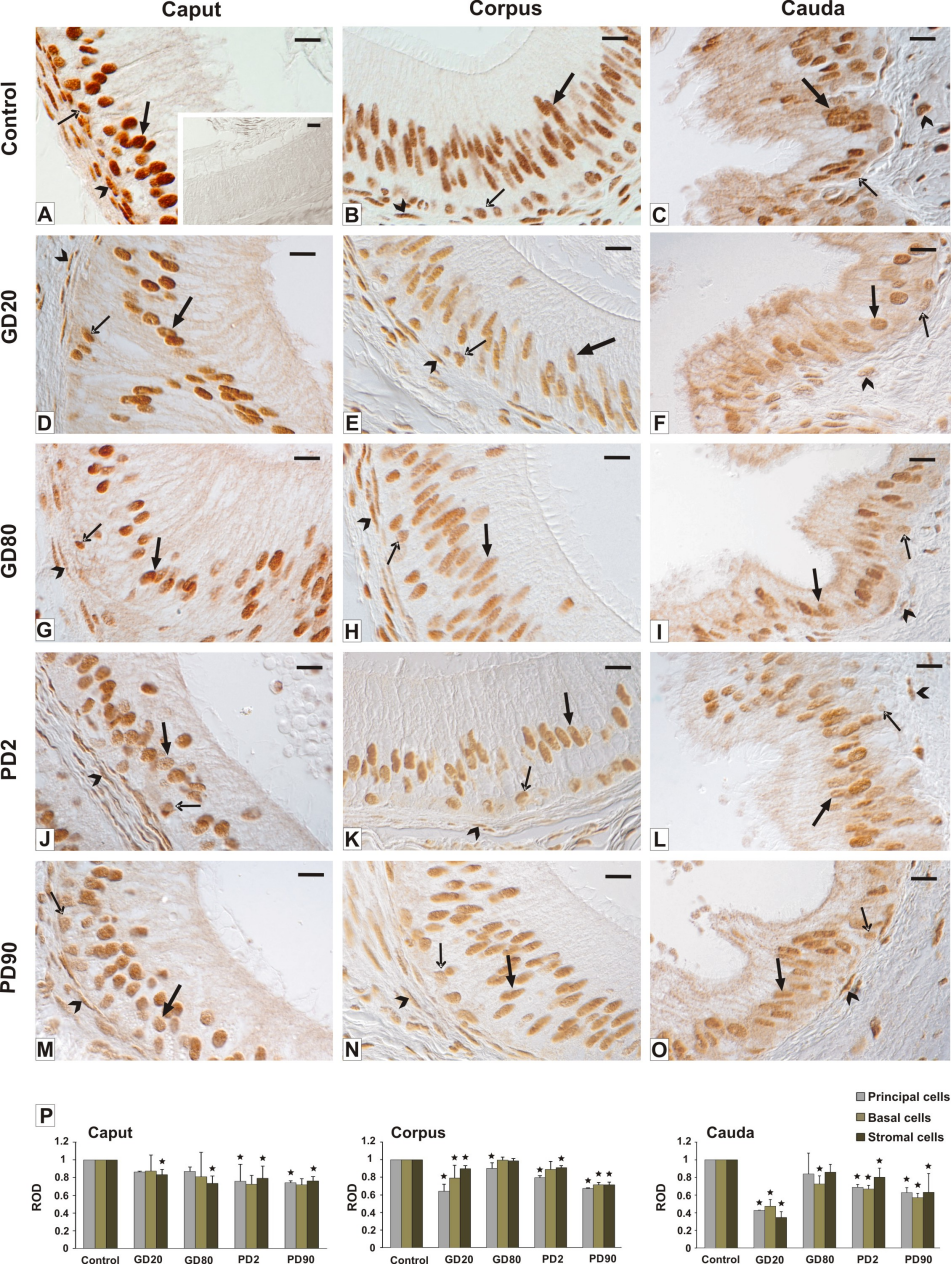
**Qualitative and quantitative analysis of immunohistochemical staining for AR**. Representative micrographs of control **(A-C) **and flutamide-treated boars **(D-O) **and a histogram of AR staining intensity expressed as relative optical density (ROD) of diaminobenzidine brown reaction products **(P)**. Caput epididymis. In the control group **(A) **very strong intensity of AR staining is seen in the nuclei of principal cells (bold arrow), moderate to strong staining is seen in basal cells (arrow) and in stromal cells (arrowhead). In flutamide-treated males moderate to strong staining is seen in the nuclei of principal cells, weak staining is visible in basal cells and stromal cells **(D, G, J, M)**. Note a slight decrease in the staining intensity in principal and basal cells, while in stromal cells a marked decrease is seen **(F, I, L, O) **Corpus epididymis. In the control group (B) strong intensity of AR staining in the nuclei of principal, basal, and stromal cells is seen. Note a marked decrease in the staining intensity in all cell types after flutamide-treatment **(E, H, K, N) **Cauda epididymis. In the control group **(C) **moderate to strong intensity of AR staining in the nuclei of principal and stromal cells and weak staining in basal cells is seen. Note a significant decrease in the staining intensity in principal and basal cells after flutamide exposure **(F, I, L, O)**. In the control tissue section, no immunostaining for ARs is observed when the incubation was performed without the primary antibody (insert in **A**). principal epithelial cells - large arrows, basal epithelial cells - arrows, stromal cells - arrowheads. Nomarski interference contrast. Scale bar represents 20 μm. Values are means ± SD. Asterisks indicate significant differences between the respective control group and groups treated with flutamide prenatally (GD20, GD80) and postnatally (PD2, PD90) (n = 3 for each group). Statistical significance: *p < 0.05.

Several types of AR-immunopositive cells were found in the epididymides of flutamide-exposed boars and control boars (Figure 5A-O). In detail, strong to very strong, exclusively nuclear staining for AR was detected in both, principal cells and basal cells of the three regions of control epididymis (Figure 5A-C). After prenatal exposure, and, in a lesser extend after postnatal exposure to flutamide, the intensity of the staining was almost similar in the principal cells of the caput, slightly reduced in both epithelial cells of the corpus and cauda, and apparently reduced in the basal cells of the cauda region (Figure 5D-O). The staining was also significantly reduced in the stromal cells surrounding the epididymal duct. Qualitative analysis of immunoreactivity for AR (Figure 5A-O) was confirmed by quantitative image analysis (Figure 5P). In flutamide-treated males statistically significant decrease in AR expression (*p *< 0.05) was found in the stromal cells of all three regions and in the principal cells of the corpus and cauda (*p *< 0.05). The AR expression was also distinctly reduced in the basal cells, however statistically significant differences (*p *< 0.05) were found mostly in the cauda, as it was noted for the principal cells. For details, see Figure 5P. No immunopositive signal for AR was found when the incubation was performed with omission of the primary antibody (insert in Figure 5A).

**Figure 6:**
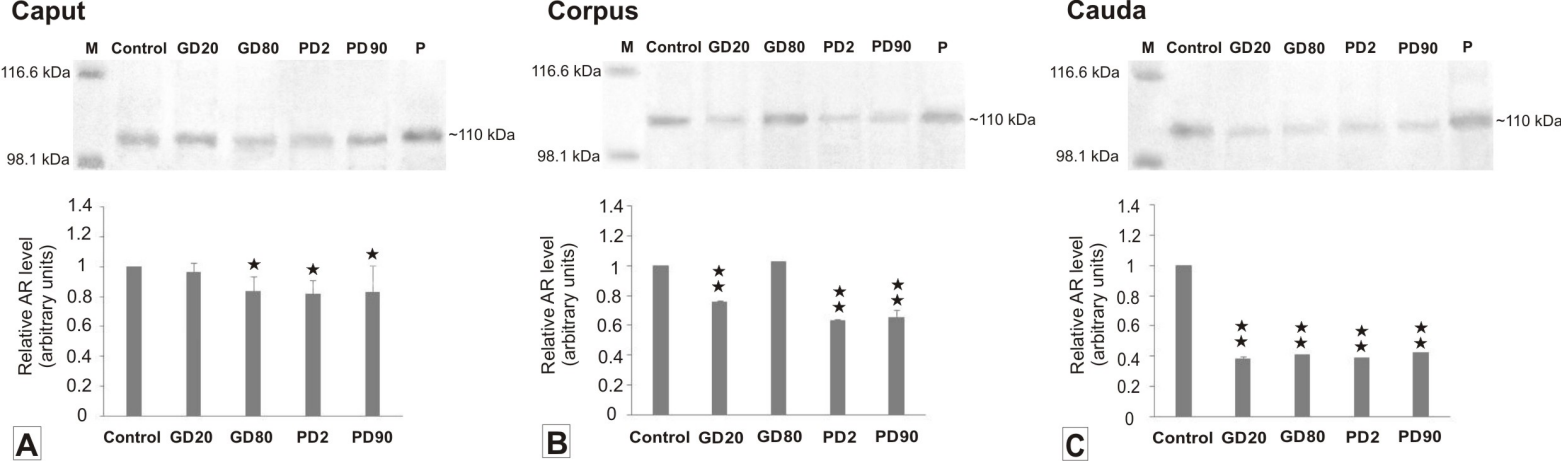
**Western blot analysis of AR protein expression levels in epididymides of control and flutamide-exposed boars**. Representative Western blot shows a band at approximately 110 kDa in epididymal samples of control and flutamide-treated groups (prenatal exposure - GD20, GD80; postnatal exposure - PD2, PD90). Lane P indicates pig prostate used as the positive control. Lane M contains prestained protein standards; the molecular weights in kDa are indicated on the left. Relative AR levels in the caput, corpus and cauda epididymides **(A, B, C) **expressed as arbitrary units. Data obtained from three separate analyses is expressed as mean ± SD (n = 3 for each group). Significant differences from control values are denoted as **p < 0.01.

Changes in the AR protein expression levels following exposure to flutamide versus the controls were assessed by Western blot analysis. As expected, a band at approximately 110 kDa was detected in epididymal homogenates of control pigs. The presence of the AR protein was also found in tissue samples of GD20, GD80, PD2, and PD90 groups (Figure 6). No changes in the level of AR were noted in the caput epididymis irrespectively of the time of flutamide exposure when the data points were compared with the control group. In contrast, statistically significant decrease in the protein level was noted in the corpus region of GD20, PD2 and PD90 males (*p *< 0.01) and in the cauda of all groups receiving flutamide (Figure 6A-C). The above data point out that early gestational and postnatal flutamide treatment had a profound, likely region-specific effect on AR protein expression at adulthood.

## Discussion

In the study herein we characterized alterations in the epididymis histology, changes in the number of apoptotic cells and changes in the expression of Cx43 and AR proteins in adult boar epididymis after prenatal and postnatal exposure to flutamide. The findings examining the long-term effects of flutamide on the mature epididymis provides new data on that reproductive organ and its function in the boar.

### Effect of flutamide on morphology of the boar epididymis

Histological examination revealed decreased height of the epididymal epithelium in the corpus region, whereas the thickness of the stroma layer enlarged significantly in the cauda. Moreover, the caudal epididymal segment of GD20 and GD80 groups exhibited less folded appearance than that of control. These results are in line with the study by Smithwick and Young [32] who reported a decrease in epithelial height and loss of stereocilia in androgen-deprived epididymis of adult chimpanzee. Interestingly, in the cauda region of PD2 and PD90 groups we observed bubble-like vesicles of sloughed cytoplasmic membranes, likely derived from stereocilia, whereas in the caput of PD2 and PD90 groups agglomeration of leukocytes characteristic of an inflammation response to tissue damage were detected. Massive leukocyte infiltration in rat epididymis as associated with suppression of testosterone production has been reported by Atanassova et al. [33]. It is possible therefore that the increased number of leukocytes, as a consequence of flutamide action, is associated with their phagocytic role in the epididymis. The presence of spermatophages has earlier been reported in the stallion epididymis [34], while the occurrence of the intra-epithelial lymphocytic population has been demonstrated in human epididymis [35].

### Effect of flutamide on apoptotic cell death along the boar epididymis

The results herein revealed a statistically significant increase (*p *< 0.05) in the number of apoptotic epithelial cells throughout the boar epididymis of GD20 group. After postnatal treatment a distinct increase in the number of TUNEL-positive cells was observed in the caput and cauda. These data indicate that the cell death could be activated by flutamide action. A region-specific difference of apoptosis in epididymis has also been observed after castration in rats [36]. It is well known that the caput epididymis requires higher androgen level to maintain its morphology and functions, thus it seems likely that more cells undergoing apoptosis might occur as a consequence of impaired androgen action. In accord, long-term apoptotic cell death process has been reported in adult rat germ cells exposed *in utero *to flutamide and vinclozolin [37,38]. According to McLachlan and co-workers [39] the resulting decrease in testosterone level and/or its action appears to be the primary cause for the negative effect on spermatogenesis. The above data allow the explanation for scarce spermatic content observed in the cauda epididymis of GD20, PD2, and PD90 groups. Moreover, inhibition of the sperm transition to the corpus epididymis was observed, probably as a result of a massive cell sloughing that formed intraluminal cell debris in the caput region of PD2 and PD90 groups. In contrast, acceleration of sperm transition through epididymis by androgen deprivation has been reported in rats exposed to several environmental contaminants [40]. It should be mentioned that any toxic insult to testicular Leydig cells causes androgen deprivation in rete testis fluid, which in consequence, can affect normal secretion of various proteins by epididymal cells. It is also likely that round cells observed in the lumen of flutamide-exposed males may derive from the testes, since a drastic decrease in testicular germ cell population was observed as a consequence of flutamide action (unpublished data). Our results are in line with the study by O'Donnell and co-workers [41] who observed appearance of round spermatids detached from seminiferous epithelium as a result of testosterone withdrawal in the rat. A role of androgens along the epididymis in creating the microenvironment necessary for maturation and storage of spermatozoa has thoroughly been analyzed by Hinton and Palladino [42]. Thus, differential response to flutamide among the cells of the various segments of the boar epididymis supports the notion that the caput, corpus and cauda are not equally dependent on androgen.

### Effect of flutamide on AR and Cx43 expression along the boar epididimis

After flutamide exposure we showed a positive staining for ARs (though of various intensity) in the principal and basal epithelial cells and in the stroma layer throughout the epididymis. This reflects the abundance of different cell types of the epididymis that are directly regulated by androgens. Whatever the cell type the staining was restricted to the cell nuclei and the pattern of the staining remained unchanged in flutamide-exposed boars. In contrast, Trybek et al. [43] reported affected staining pattern in finasteride-treated rats; ARs were localized to the cytoplasm of epithelial epididymal cells. Moreover, we noted a significant decrease in the staining intensity in both epithelial cells of the corpus and cauda regions. On the other hand, lack of significant alterations in the AR protein level within the caput of flutamide-treated boars could be explained by too high androgen level in this region, which could not effectively be blocked by the flutamide. Turner et al. [44] reported that the total androgen level in the lumen of the caput epididymidis is 6-fold higher than that in the cauda epididymis. Furthermore, the intensity of the staining and the number of immunopositive cells in the stroma layer were reduced from the caput to cauda regions when compared to the controls. Also the relative AR level was significantly lower (*p *< 0.01) in the corpus and cauda segments of flutamide-exposed boars. This corroborates a study by Zhu et al. [45] who demonstrated a subsequent decline of AR levels in stromal cells after azaline treatment. It seems likely therefore that the stromal cells from the distal regions of the epididymis are more sensitive than those of the caput to flutamide action. Concomitantly, exposure to flutamide (GD20 and PD2) induced a significant decrease (*p *< 0.05) in Cx43 expression in the stromal cells of the corpus and cauda regions that corresponded with a distinct reduction (*p *< 0.01) of the relative Cx43 protein expression levels in the cauda. It gives rationale that early gestational exposure to flutamide and its neonatal administration have a profound, mostly segment-specific effect on the stromal cells. Abundant intercellular communication in the stroma may be important in expelling spermatozoa from the cauda epididymis at the time of ejaculation as previously suggested by Dufresne et al. [46]. Therefore, a significant reduction in the expression of Cx43 in these cells after flutamide treatment with concomitant lack of the signal in the stromal cells suggests flutamide as targeting Cx43 protein and affecting the process of epididymal microenvironment formations. On the other hand, abundant expression of Cx43 between the stromal cells of the control boar epididymis indicates the role of gap junctional communication in normal contraction of epididymal duct. It seems likely therefore that drastic decrease in the expression of Cx43 observed in the stromal cells after exposure to flutamide at GD20 and PD2 could be responsible for altered transit of spermatozoa from the corpus to cauda epididymis. This indicates that the expression of Cx43 protein in the stroma of adult pigs could be regulated, among other factors, by androgens. These results are in line with the study by the group of Cyr [47] who has proposed a crucial role for Cx43 to rat epididymal function. They showed altered Cx43 expression in orchidectomized rats what suggests that androgen may regulate the gap junction protein expression. In another study, Cyr and co-workers demonstrated that thyroid hormones preferentially regulate the connexin expression in proximal segments of the epididymis but not in the cauda region [48]. Interestingly, in GD20 and GD80 groups we observed a high expression of Cx43 protein between adjacent principal cells at their apical or lateral margins in the initial segment, not in the caput region. In the latter, small punctate binding sites for Cx43 were found only at the base of the epithelium. Such localization is consistent with that observed in the stallion and Egyptian water buffalo epididymides [25,49], respectively. According to Cyr et al. [47] the regulation of Cx43 in the epithelium of the initial epididymal segment indicates that androgens regulate the targeting of Cx43 towards specific cell-to-cell interface. Based on recent studies, however, Pointis and co-workers [50] stated that the reason why androgens preferentially regulate connexin expression in different regions of the epididymis still remains unknown.

## Conclusions

Concerning our results, it seems likely that the mechanisms that allow flutamide to interfere with numerous physiological processes by blocking androgen receptors are fully developed at adulthood. This seems to be in line with earlier studies on rats demonstrating the specificity of epididymal secretion as progressively established with age [51,52]. Flutamide, by blocking the androgen action, is responsible for an impairment of the microenvironment created by epididymal cells for sperm maturation and their storage. A decrease in AR protein levels results in altered androgen signaling which may cause disturbances in androgen-dependent processes including Cx43 (de)regulation, however, we can not exclude the possibility that in response to flutamide decreased Cx43 expression may represent one mechanism responsible for functional disturbance of the boar epididymis. Summing it up, the results indicate that early developmental window and the neonatal period of time are the most critical for flutamide-induced changes later in life of the boar.

## Competing interests

The authors declare that they have no competing interests.

## Authors' contributions

ML carried out qualitative and quantitative immunochemistry, Western blotting and performed the statistical analysis; IK-S performed the TUNEL assay and helped to draft the manuscript; MK-B participated in the sequence alignment and analyzed data; KC and DZ (students) carried out histology and provided technical help; BB designed the study and drafted the manuscript. All authors read and approved the final manuscript.

## Authors' information

The results reported herein are part of ML's PhD studies performed at the Jagiellonian University of Krakow for a doctor of philosophy degree.
